# Essential Oil of Fractionated Oregano as Motility Inhibitor of Bacteria Associated with Urinary Tract Infections

**DOI:** 10.3390/antibiotics13070665

**Published:** 2024-07-18

**Authors:** Eduardo Sánchez García, Cynthia Torres-Alvarez, Elías G. Morales Sosa, Mariana Pimentel-González, Licet Villarreal Treviño, Carlos Abel Amaya Guerra, Sandra Castillo, José Rodríguez Rodríguez

**Affiliations:** 1 Facultad de Ciencias Biológicas, Universidad Autónoma de Nuevo León, Av. Universidad s/n, Ciudad Universitaria, San Nicolás de los Garza 66455, NL, Mexico; eduardo.sanchezgrc@uanl.edu.mx (E.S.G.); elias.moralesso@uanl.edu.mx (E.G.M.S.); mariana.pimentelg@uanl.edu.mx (M.P.-G.); licet.villarrealtr@uanl.edu.mx (L.V.T.); carlos.amayagr@uanl.edu.mx (C.A.A.G.); 2 Facultad de Agronomía, Universidad Autónoma de Nuevo León, Francisco Villa s/n, Ex-Hacienda “El Canadá”, General Escobedo 66050, NL, Mexico; cynthia.torresalvr@uanl.edu.mx; 3 Tecnológico de Monterrey, Escuela de Ingeniería y Ciencias, Ave. Eugenio Garza Sada 2501, Monterrey 64849, NL, Mexico

**Keywords:** antibiotic resistance, antibacterial activity, essential oils, swimming, swarm motility

## Abstract

In this research, several analyses were carried out on concentrated fractions of Mexican oregano essential oil (*Poliomintha longiflora* Gray) in order to determine its ability to inhibit the growth and the motility of *Escherichia coli* (swimming), *Pseudomonas aeruginosa* (swimming), and *Proteus vulgaris* (swarming); these Gram-negative bacteria associated with urinary tract infections are motile due to the presence of flagella, which is considered an important virulence factor that favors their motility when trying to reach the target organ and cause an infection. Also, the resistance pattern to antibiotics of each strain was determined. The results showed resistance pattern (8 out of 12 antibiotics tested) for *P. aureginosa*, while *E. coli* and *P. vulgaris* were resistant to 4 antibiotics out of the 12 tested. On the other hand, fractionated oregano caused an inhibition of growth and a reduction in motility, varying between fractions and among bacteria. Fraction 4 showed major growth reduction, with MBC values ranging from 0.002 to 23.7 mg/mL. Treatment with fractionated oregano (F1, F2, F3, F4) reduced the motility by 92–81% for *P. vulgaris*, 90–83% for *E. coli*, and 100–8.9% for *P. aeruginosa*. These results demonstrated a higher performance with a lower application dose due to its high content of Carvacrol and Thymol; unlike other concentrated fractions, this synergy of oxygenated monoterpenes may cause greater antimicrobial activity.

## 1. Introduction

The implementation of natural compounds in both pharmaceutical and food products is very relevant today since bacterial resistance to antibiotics has evolved and has become a matter of concern [1,2]. The World Health Organization (WHO) has warned that in the year 2050, the main reason for deaths will be caused by drug-resistant bacteria, mainly due to the misuse of medicines, which promotes the adaptation of microorganisms to new mechanisms of drug action [3,4]. The increase in antibiotic-resistant strains associated with infections demand novel therapeutic strategies for their inactivation [5]. Virulence plays a critical role in the infection process and enables the pathogen to damage the host [6]. Targeting microbial virulence instead of survival seems to be a good strategy due to the low selective pressure for the development of bacterial resistance [7,8]. Bacterial motility is considered an important virulence factor since this characteristic is crucial for approaching target organs and plays a key role in bacterial colonization and subsequent biofilm formation [6,9]. *Escherichia coli*, *Proteus vulgaris*, and *Pseudomonas aeruginosa* are bacteria involved in urinary tract infections [10,11]; they show different resistance patterns and reflect changes over the years [12,13,14]. For example, catheter-related infection of the urinary tract and upcoming biofilm formation by *Proteus* species are promoted by the swarming activity of these microorganisms [15,16]. In order to find an appropriate alternative therapy, it is important to describe the resistance pattern of implicated bacteria [11,12] and the virulence susceptibility to natural products as well [6,17]. On the other hand, oregano has been recognized for many years as medicinal due to its antimicrobial activity; however, these characteristics may vary, depending on the species and type of crop, which will influence the compounds present [18,19,20]. Oregano essential oil (OEO) is a complex mixture of volatile compounds and is considered a safe, natural alternative (GRAS) by the WHO [21,22]. The OEO possesses antioxidant and antibacterial activities probably conferred by the presence of Thymol and Carvacrol; however, synergistic activity has been observed when combined with other oxygenated monoterpenes [17,19]. In this sense, the antimicrobial and/or antioxidant effect of OEO may vary depending on the chemical composition governed not only for the qualitative aspect but the quantitative aspect: proportions and amounts of compounds. Although various investigations have been carried out to evaluate the antimicrobial activity of oregano, very few have involved the Mexican species *Poliomintha longiflora.* Additionally, various concentrated fractions have been obtained from certain essential oils, including the essential oil obtained from *Poliomintha longiflora*; these fractions have shown important differences in their composition as well as in their antimicrobial effect against different bacteria [20,23]. Even though anti-motility studies have been carried out on several bacteria, most report the effects caused by pure compounds and/or essential oils [16,24]. There are no reports of anti-motility effects regarding these concentrated fractions. In an effort to avoid the development of antimicrobial resistance, these studies acquire a significant relevance. For the previously mentioned reasons, this study states as a hypothesis that the different fractions of OEO possess different levels of antimicrobial and anti-motility activity against these motile bacteria.

## 2. Results

### 2.1. Obtention of the Fractionated Oregano Essential Oil (OEO)

Fractionated oregano was obtained through fractional distillation. Four fractions and a residual oil were collected; physical properties—color, odor, and specific gravity (g/mL)—are reported on Table 1. The color varied between the fractions collected, as well as the odor.

**Figure 1:**
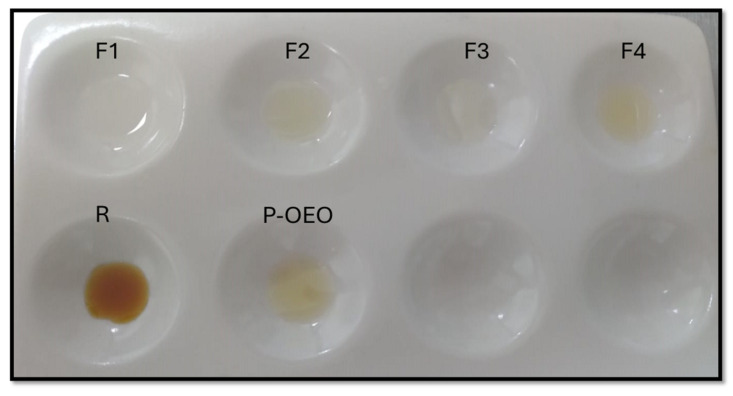
Fractions collected through fractional distillation. Fraction 1 (F1), Fraction 2 (F2), Fraction 3 (F3), Fraction 4 (F4), Residual (R), and P-OEO.

F1 and F3 showed a colorless and very faint color, respectively, while F4 presented a very similar color to P-OEO. On the other hand, F2 had an intermediate yellow color, while residual oil presented a strong brown color (Figure 1). It is difficult to characterize the odor of each one of the fractions obtained; even though the odor is classified as strong, soft and intermediate, there are different aromatic notes in each fraction, which are difficult to describe. Additionally, all fractions presented a pleasant aroma.

#### Gas Chromatography-Mass Spectrometry (GC-MS)

The chemical profile of each fraction was performed and analyzed by GC-MS. The profile of organic compounds for each fraction is shown on Table 2. These compounds are classified as monoterpene hydrocarbons (MHs), oxygenated monoterpenes (OMs), and sesquiterpenes hydrocarbons (SeHs). According to the results, there are marked differences in both the composition and the amount of each compound for each fraction. F1 and F2 had MHs as the majority compounds (92.12 and 83.91, respectively) and OMs in a low proportion (7.81 and 10.12, respectively); the SeH compounds were not detected for these two fractions. On the contrary, in F3 and P-OEO, the three groups of compounds were detected with a majority of MHs (48.79 and 38.60, respectively) and OMs (41.53 and 52.19, respectively) while SeHs were present in a low proportion (6.52 and 9.21, respectively). On the other hand, in F4 and residual (R), OMs were found as the majority compounds (84.77 and 70.14) and SeHs were present in a low proportion (13.03 and 22.13), while MHs were not detected (Table 2). These differences in composition are very important because of the properties associated with each fraction.

### 2.2. Susceptibility Characterization of Strains

The study regarding the susceptibility of commercial antibiotics to bacteria of interest was carried out according to recommended CLSI standards. The results showed a critical resistance for *P. aeruginosa* since it was resistant to 8 out of the 12 antibiotics tested. The resistance was observed for AM, CF, CFX, DC, NET, NF, PE, and SXT. The susceptibility of *P. aeruginosa* was observed only for four antibiotics: AK, CL, CTX, and GE.

In the case of *E. coli* and *P. vulgaris*, they exhibited some variability regarding their susceptibility when compared with *P. aeruginosa*, which showed a resistance to 4 out of the 12 antibiotics tested.

Even though these two bacteria were resistant or susceptible to the same number of antibiotics, they presented variability in the type of antibiotic. Resistance in *E. coli* was observed for AM, CF, DC and PE, while *P. vulgaris* presented resistance for CFX, CL, PE, and SXT. The results are presented in Table 3.

### 2.3. Preliminary Antimicrobial Activity of Fractionated Oregano

**Figure 2:**
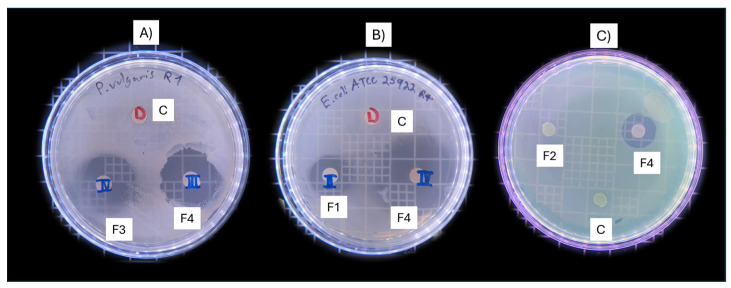
Preliminary antimicrobial activity of fractionated oregano. (**A**) Effect of F3 and F4 on *P. vulgaris.* (**B**) Effect of F1 and F4 on *E. coli*. (**C**) Effect of F2 and F4 on *P. aeruginosa*. Fractions 1–4 (F1, F2, F3, F4). Control (C).

The antimicrobial activity of Mexican fractionated oregano against *E. coli*, *P. vulgaris*, and *P. aeruginosa* varied among strains; different fractions of oregano showed differences in inhibition zones (Figure 2).

According to the results obtained, the P-OEO and F4 demonstrated greater antimicrobial performance compared with Fractions 1, 2, and 3, and Residual (F1, F2, F3, R), with the exception of *P. aeruginosa*. The inhibition zone diameter of P-OEO ranged from 3.3 to 2.3 for *E. coli* and *P. vulgaris*, respectively. In contrast, *P. aeruginosa* showed a resistance with a diameter of 0.7 cm (Table 4). Fraction 4 showed the best antimicrobial activity with inhibition zones ranging from 3.8 to 1.4 cm, with *E. coli* being more susceptible than *P. vulgaris* (2.4 cm) and *P. aeruginosa* (1.4 cm). Fraction 3 and R showed similar results and better activity than F1 and F2. In general, *P. aeruginosa* exhibited more resistance than the other bacterial strains (Figure 2 and Table 4).

### 2.4. Minimum Bactericidal Concentration (MBC)

The MBC was determined for the fractionated oregano against the three motile strains (Table 5). According to the results, *P. aeruginosa* showed greater resistance with values of 21.2 to 23.7 mg/mL and did not show inhibition (for the concentrations tested) when treated with F1 and F2. On the other hand, *E. coli* showed more susceptibility for F3, F4, P-OEO, and R, with low MBC values ranging from 0.1 to <0.002 mg/mL; higher values of MBC were observed for F1 (22.5 mg/mL), while F2 had a MBC value of 2.1 mg/mL. Finally, *P. vulgaris* was found to be more susceptible, with MBC values ranging from 0.002 to 1.1 mg/mL. In general, the fractions with the best antimicrobial activity were F3, F4, and Residual. Significant differences were evidenced between F4 compared with P-OEO and residual only for *P. vulgaris* (Table 5).

### 2.5. Anti-Motility Effect of Fractionated Oregano

**Figure 3:**
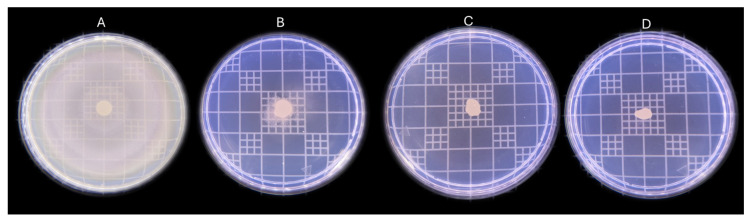
Anti-motility effect of fractionated oregano against *P. vulgaris*. (**A**) Control: without fractionated oregano, (**B**) Fraction 4, (**C**) Residual oil, (**D**) P-OEO.

**Figure 4:**
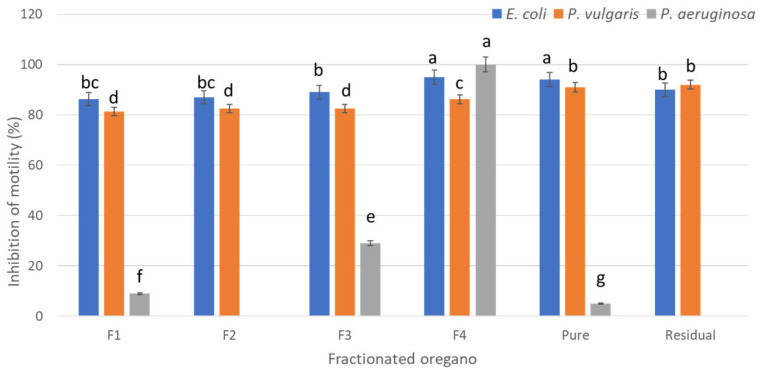
Inhibition of bacterial motility after incubation with different fractions of oregano. The concentration added was the 50% of the MBC. Different letters are significant differences. Error bars represent standard deviation.

The anti-motility effect of each fraction was tested against bacteria in a semi-solid agar supplemented with 50% of the MBC of each fraction or P-OEO (Figure 3). This concentration was used since it did not cause a decrease in the microbial population. Even though no significant difference in the MBC of *P. aeruginosa* for F4 and P-OEO (Table 5) was found, the effect on motility was very different for both: F4 completely inhibited the motility for *P. aeruginosa*, while P-OEO presented an inhibition of only 8.9% (Figure 4). Although F1 did not show an MBC, when an anti-motility test was carried out, F1 evidenced 10% of the motility inhibition for *P. aeruginosa*. On the other hand, F3 had an inhibition of 32%, while F2 and Residual did not exert an inhibition on *P. aeruginosa*. In contrast, the motility inhibition of *E. coli* and *P. vulgaris* was evidenced for all the fractions tested. Values of motility inhibition ranged from 81 to 96%; once again, F4 and P-OEO had the best anti-motility activity (Figure 4). Surprisingly, the residual oil had an anti-motility effect against *P. vulgaris* comparable to that of P-OEO, both being significantly greater than F4 (Figure 3 and Figure 4).

### 2.6. Principal Component Analysis

**Figure 5:**
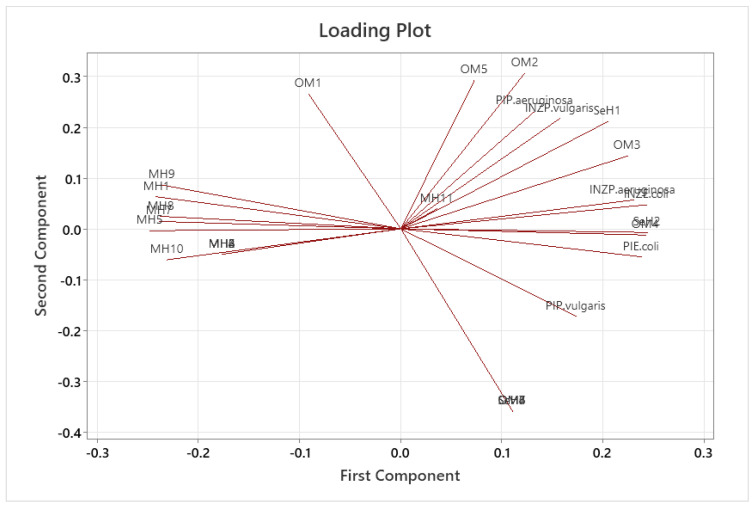
Loading plot that presents the distributions of the variables in the first and second principal components.

The principal component analysis is shown in Figure 5, in which the loading plot allowed us to establish the correlation of the organic compounds detected in each fraction (F1-4, R, and P-OEO) with the percentages of motility inhibition and the growth inhibition zones in *E. coli*, *P. vulgaris*, and *P. aeruginosa*; the formation of two components that can be observed explains 88.1% of the data variability. Groups of compounds can be observed to correlate with each other; in a group, the compounds labeled OM2, OM3, OM4, and OM5 are correlated—that is, the oxygenated monoterpenes. In the same way, another group of compounds with the labels MH1, MH5, MH9, MH10, MH8, and MH3 corresponds to monoterpene hydrocarbons. It is observed that Eucalyptol (OM1) is placed somewhat distantly from the group of oxygenated monoterpenes, just like the monoterpene hydrocarbon α-Terpinene (MH11), which has a tendency to group with the oxygenated monoterpenes.

It is clearly highlighted that the biological activities were evaluated; the percentage of immobility (PI) and antimicrobial activity by inhibition zone (INZ) for the bacteria *E.coli*, *P. vulgaris*, and *P. aeruginosa* have a positive correlation at their highest magnitude with the oxygenated monoterpenes when they are together: Terpinen-4-ol (OM2), Thymol (OM3), Carvacrol (OM4), and Thymyl methyl ether (OM5). On the other hand, the group of monoterpene hydrocarbons shows a negative correlation with the previously mentioned biological activities.

**Figure 6:**
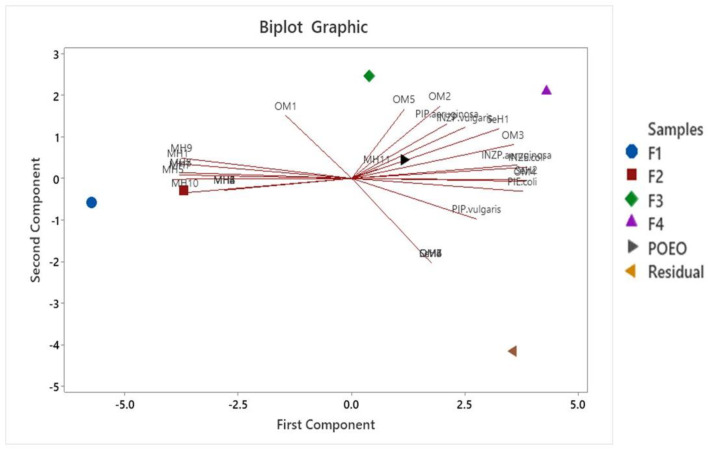
Biplot of variables and type of samples in the multivariate analysis.

Figure 6 shows the Biplot graph—that is, a diagram where the distribution of the samples (Fractions) is observed in the same factorial plane where the coordinates correspond to the loading plot graph of the principal components.

The Biplot graph shows a positive correlation of Fractions 3 and 4 and P-OEO, with the group of oxygenated monoterpenes, and a negative correlation with the monoterpene hydrocarbons, in addition to the fact that the opposite occurs in Fractions 1 and 2.

## 3. Discussion

Urinary tract infections (UTIs) are very common. About 150 million cases are reported around the world, and complicated cases are increasing due to the antibiotic resistance of the bacteria associated with these infections [4,12]. The appearance of multidrug-resistant strains isolated from patients is increasing and is therefore generating concern among the medical community. This has led to the search for new control alternatives for these microorganisms; essential oils have received great attention for this effect [26,27]. It is known that oregano essential oil has antimicrobial properties; however, these effects may vary depending on the type of oregano and climatic conditions during cultivation [18,19]. It has been demonstrated that fractionated oregano possesses different bactericidal levels; in addition, each fraction obtained varied in its composition, making it an increasingly laudable alternative for coadjutant application [20,24,26,27,28]. Although the antimicrobial effects are reported, the effects of these fractions on virulence factors such as bacterial motility are not known. According to our results, the strains used in this study are multi-drug-resistant, with *P. aeruginosa* being the most antibiotic-resistant bacteria with susceptibility only to four (AK, CTX, CL, and GE) out of the twelve antibiotics tested. On the other hand, *E. coli* and *P. vulgaris* showed resistance to four antibiotics out of the twelve tested (Table 3). Our results are in accordance with Mustika et al. [29], who investigated the multi-resistance of 41 isolated strains of *E. coli* in cattle in slaughterhouses. They found 29.27% resistance to amoxicillin, 24.39% to tetracycline, 7.32% to ciprofloxacin, 2.44% to gentamicin, and 2.44% to ceftazidime. It is worth mentioning that some issues that influence drug resistance could be the use of antibiotics as food additives in livestock, for either preventing or curing diseases. Regarding this issue, Lu et al. [30] reported the resistance showed by *Pseudomonas* isolates to 14 antibiotics tested; they were susceptible only to imipenem (IPM). Furthermore, Bilal et al. [31] conducted a study with samples from patients suffering from urinary tract infections; they isolated *P. vulgaris* and found that 94% of the strains had resistance to ampicillin, chloramphenicol and tigecycline, 88% to cefotaxime, 76% to ciprofloxacin and nitrofurantoin, and 59% to amikacin. They argued that strains isolated may present variations in the antibiotic resistance pattern due to the frequency of their use in the different patients. According to recent investigations, natural plant-derived compounds represent a promising subject because they are beneficial in some ways: firstly, the anti-virulence action could support the immune response, which in turn could stop the infection before it starts; in this way, the potential drug resistance issues are avoided [6,8,26]. In this investigation, the antimicrobial and anti-motility properties of fractionated Mexican oregano were evaluated; of all the oregano oil fractions studied, F4 presented the highest antimicrobial activity, with inhibition zones ranging from 1.4 to 3.8 cm, while the MBC observed ranged from 0.002 to 23.7 mg/mL. This strong activity shown by F4 was followed by P-OEO and R; in contrast, F1, F2, and F3 presented lower activities. Typically, the zone of inhibition and the MBC are inversely linked. If the microbe is highly susceptible to the antimicrobial agent, the MBC will be lower, and the zone of inhibition will be larger [32]. This behavior was evident in our results, in which those larger inhibition zones had a lower MBC. Our results are similar to those reported by Pérez-Delgado et al. [33], who evaluated the antibacterial activity of ethanolic extracts of *Origanum vulgare* against *Pseudomonas aeruginosa* and *Escherichia coli*; they reported average inhibition halo sizes of 13.3 mm for *P. aeruginosa* and 12.5 mm for *E.coli*. Also, Alkhafaji and Jayashankar [34] studied the antibacterial activity of oregano oil against urinary tract pathogens, including *E. coli* and *P. aeruginosa*, through inhibition zones using the diffusion method; particularly for *E. coli*, they found inhibition zones of 2.9 cm, while it was 2.7 cm for *P. aeruginosa*. Both authors suggest that this biological activity could be attributed to the abundance of oxygenated monoterpenes and phenolic compounds such as Carvacrol, Thymol, and *p*-Cymene. Similarly, Dias et al. [35] investigated the therapeutic use of essential oils from *Lavandula luisieri* and *Cymbopogon citratus* (known to contain higher amounts of oxygenated monoterpenes) against fungus involved in dermatophytosis; they suggested an association of the oxygenated monoterpenes compounds with this antifungal activity. Additionally, Janani et al. [36] reported oxygenated monoterpene Carvacrol as the major constituent of *Oreganum vulgare* and strong antibacterial activity against *Enterococcus fecalis*; they attribute the antimicrobial activity to the presence of this last compound.

Table 1 shows the composition of the oregano oil fractions used in this study. F4 had the highest number of oxygenated monoterpenes (84.77%) followed by R (70.14%), and P-OEO (52.19%); Thymol and Carvacrol are the major compounds in all of them (F4-21.17/60.23%, R-10.42/48.54%, P-OEO-13.53/34.09%, respectively). On the other hand, low proportions of these compounds were detected in F1, F2, and F3. According to Mora-Zúñiga et al. [19], the presence of Thymol and Carvacrol plays an important role in antimicrobial activity. Likewise, it has been observed that a synergistic behavior occurs when these two latter compounds are combined or when they are in combination with other monoterpenes [20]. Since these two compounds have been reported to be responsible for antimicrobial activity [33,34,35,36] and are also the majority compounds present in F4, R, and P-OEO, the bactericidal effect could be attributed to these compounds.

Virulence enables the pathogen to infect and damage the host; targeting virulence rather that killing bacteria represents an alternative with promising applications due to the low selection pressure that reduces the chances of developing drug resistance—hence, anti-virulence drugs have acquired special importance [17,24,37]. Plants are valuable resources for the development of novelty anti-virulence drugs; likewise, essential oils seem to be the predominant anti-virulence components of spices [5,24]. As a result of this, research that provides a better understanding of those properties is crucial [1,8].

Bacterial motility is a very important virulence factor since it is associated with quorum sensing system and biofilm formation; it also plays a crucial role in intestinal colonization and invasion [38,39]. Flagella-regulated motility is critical for the pathogenesis of *P. aeruginosa*, *P. vulgaris*, and *E. coli* [24,40]. Our results evidenced that fractionated oregano has an anti-motility effect on *E. coli*, *P. vulgaris*, and *P. aeruginosa*. This effect varied among fractions and strains. The major anti-motility activity in *P. aeruginosa* was observed for F4, while for *E. coli* and *P. vulgaris*, all the fractions showed motility inhibition above 80%. This effect was evidenced also for F1 and F2, although these last two fractions showed significantly lower antimicrobial activity than the others. Despite the fact that in recent reports [33,34,35,36] the antimicrobial activity of oregano essential oil has been attributed to oxygenated monoterpenes (OMs), mainly due to the presence and amount of Carvacrol and Thymol, the anti-motility activity seems to be influenced by the synergy of other OM compounds. The results of the principal component analyses evidenced that the antimicrobial and anti-motility activities detected are correlated with OM 2, 3, 4, and 5 (Terpinen-4-ol, Thymol, Carvacrol and Thymyl methyl ether, respectively); however, there are two compounds separated from their groups: OM1 (Eucalyptol) and MH11 (α-Terpinene). Since OM1 is present in both F1 and F2, and these two fractions had lower antimicrobial activity but great anti-motility activity, we could assume that anti-motility activity in these two fractions is influenced by the synergy that could occur when OM1 is combined with OM3 and OM4. It should be noted that there are other molecular factors specific to the bacteria that are not well understood; therefore, there is a variability in the effectiveness of essential oils, such as the case of *P. aeruginosa* [24].

## 4. Materials and Methods

### 4.1. Obtention and Physical Chemistry Characterization of the Fractionated Oregano Essential Oil (OEO)

The P-OEO (pure oregano essential oil) was obtained by steam distillation [20,25] and was provided by the OR-LAG company. The plant material (*Poliomintha longiflora* Gray) was collected in a mountainous region where the Mexican states of Coahuila, Durango, and Zacatecas converge. The coordinates are 24°55″ latitude (north) and 103°10″ longitude (west). The plant was collected 15 days after precipitation. The phenological stage of the plant at the time of harvest was during and 10 days after flowering. The composition of the vegetal material used was leaf, flower, and stem in a proportion of 90:9:1, respectively. The specimen was identified and deposited in the herbarium of the Facultad de Ciencias Biológicas. In order to obtain the fractionated OEO, a fractional distillation system was established according to the method mentioned by Rostro-Alanis et al. [20], with some modifications. Fraction 1 started to distill at a temperature of 82 °C, while the last fraction started to distill at 140 °C. Finally, undistilled oil (Residual) was obtained. At the end of the process, five fractions identified as Fraction 1 (F1), Fraction 2 (F2), Fraction 3 (F3), Fraction 4 (F4), and Residual oil (R) were obtained. After that, the fractionated oregano was stored at refrigeration temperature (4 °C) in dark jars in order to be protected from light and be used for further analyses. Pure OEO (P-OEO) was used as a point of comparison.

### 4.2. Gas Chromatography-Mass Spectrometry (GC-MS)

The analyses of the P-OEO, its fractions (F1–F4), and the residual were performed with slight modifications according to the method mentioned by Rostro-Alanis et al. and Asensio et al. [20,25]. Briefly, a 1 μL aliquot of the extracts was automatically injected into a gas chromatograph and mass spectrometer (Perkin Elmer Clarus 690/SQ 8T, PerkinElmer, Waltham MA, USA). GC analyses were performed on a TG-5 MS capillary column (30 m × 0.25 mm × 0.25 μm) with helium as a carrier gas at a flow rate of 1 mL/min. The initial oven temperature was set at 70 °C for 2 min. Then, its temperature was increased by 10 °C per minute until it reached 200 °C; it was held at that temperature for 5 min. A second heating ramp was used, and its temperature was increased by 15 °C per minute until it reached 310 °C; it was held at that temperature for 5 min. The total execution time was 32.33 min. The injector temperature was set at 256 °C. The parameters for MS were as follows: ion source EI, electron energy 70 eV, quadrupole temperature 210 °C, interface temperature 210 °C, and *m*/*z* = 30–550 amu. The corresponding peak was identified by the mass spectral library, NIST Library Copyright ©️ 1998 Perkin Elmer Corporation (Waltham, MA, USA).

### 4.3. Bacterial Strains and Culture Conditions

For this study, three Gram-negative and motile bacterial strains were selected. *Escherichia coli* ATCC 25922 was acquired from the ATCC collection; the *Proteus vulgaris* and *Pseudomonas aeruginosa* strains (isolated) were donated by the General Microbiology Laboratory, located at the Facultad de Ciencias Biológicas, Universidad Autónoma de Nuevo León (UANL). These last two strains were isolated from urine of infected patients. For the tests, they were kept in a slant agar with brain and heart infusion medium (BHI) and refrigerated until use. To activate the strain, a loopful of a culture was taken and placed in fresh sterile MH broth, and it was then incubated for 18 h. The activated cultures were then adjusted to 0.5 McFarland (≈10^8^) for further analyses [41].

### 4.4. Preparation of Oil-Working Solutions

Because the essential oils are hydrophobic, oil working solutions for each fraction (F1, F2, F3, F4, Residual, and P-OEO) were prepared for antimicrobial tests. Dimethyl sulfoxide DMSO (Sigma-Aldrich, Naucalpan de Juárez, Mexico) was used as a solvent. For this, 400 μL of each fraction was mixed with 600 μL of DMSO [13]. The oil working solution was vortexed and refrigerated in the absence of light until use.

### 4.5. Characterization of Antibiotic Susceptibility of Strains

For this assay, susceptibility tests with different antibiotics against each bacterial strain were carried out according to Kirby–Bauer technique mentioned by Ortiz et al. [42] and Yao et al. [43]. This assay was conducted following the standards established by the Clinical and Laboratory Standards Institute (CLSI). Briefly, an aliquot (100 μL) of fresh cultures adjusted to McFarland standard (0.5 ≈ 10^8^) was seeded by extension in MH agar plates. After that, disks containing antibiotics (PT-36 Multibac I.D. Gram-negatives México) were placed on the agar and incubated (35 °C/24 h). After the incubation time, the inhibition zones surrounding each disk were measured with a caliper tool. The antibiotics used were Amikacin (AK, 30 mg), Ampicillin (AM, 30 mg), Cephalotin (CF, 30 mg), Cefotaxime (CFX, 30 mg), Dicloxacillin (DC, 1 mg), Ceftriaxone (CTX, 30 mg), Chloramphenicol (CL, 30 mg), Gentamicin (GE, 10 mg), Netilmicin (NET 30 mg), Nitrofurantoin (NF, 300 mg), Penicillin (PE, 10 U), and Sulfamethoxazole and Trimethoprim (STX, 25 mg). Susceptibility was determined according to the manufacturer’s instructions, in which three criteria were established according to the recommendations of CLSI: Resistant (R), Susceptible (S) or Intermediate (I).

### 4.6. Preliminary Antimicrobial Activity of Fractionated Oregano

This assay was performed by a disk diffusion assay according to the method reported by Mora-Zuñiga et al. [19] and Carović-Stanko et al. [44] with some modifications. First, 100 μL of each bacterial culture strain adjusted to 0.5 McFarland standard (1 × 10^8^ UFC/mL) was inoculated on MH agar plates. The inoculum was spread over the surface with a Drigalsky spreader. Next, filter paper discs (6 mm in diameter) previously impregnated with 10 μL of each fraction (F1, F2, F3, F4, and R) were placed in the agar surface. The completely pure OEO (P-OEO) was used as a point of comparison. After that, the agar plates were incubated at 37 °C for 24 h. The antibacterial effect was evidenced by the presence of inhibition zones surrounding the disks. The diameters of inhibition were recorded in centimeters with a caliper tool.

### 4.7. Minimum Bactericidal Concentration (MBC) and Subinhibitory Concentrations

The minimum bactericidal concentration was obtained with microdilution method according to Lazou and Chaintoutis [45] and Man et al. [13] with some modifications. Briefly, each well of 96-well plates (Corning, Costar, Cambridge, MA, USA) was filled with a volume of 180 µL of sterile MH broth. After that, 20 µL of each oil working solution was placed in the first column. Serial dilutions in each row were conducted and immediately inoculated with the bacteria (1%, adjusted at 0.5 on the Mc Farland scale). The plates were incubated at 37 °C for 24 h. After that time, 20 µL was taken from each well and spot-inoculated onto MH agar plates, which were incubated in the same conditions mentioned above. Once the incubation was completed, the MBC was determined as the lowest concentration that completely inhibited microbial growth. On the other hand, the subinhibitory concentration was defined as a concentration less than MBC that did not cause a decrease in the bacterial population [46]. The subinhibitory concentration that presented a minimum bacterial count of 1 × 10^6^ CFU/mL was selected for further analyses. Respective controls were conducted for each assay.

### 4.8. Effect of Essential Oils on Motility

The effect on the bacterial motility of subinhibitory concentrations of fractionated OEO was evaluated. Semisolid agar plates were prepared with 0.4% MH agar for *Pseudomonas aeruginosa*, 0.5% for *Escherichia coli* ATCC 25922, and 1.5% for *Proteus vulgaris*. Additionally, fractionated OEO was added to the prepared medium at a concentration of 50% of the MBC [46]. Then, 10 µL of previously activated bacteriological culture (37 °C/24 h) was placed in the center of semisolid agar plates [46,47]. Plates were incubated for 24 h at 37 °C. Motility was measured in centimeters (cm) to obtain the migration diameter. Results were expressed as percentage of reduction in motility [6,46,47]. Bacterial migration without fractionated OEO and plates containing DMSO were used as controls and represent 100% of motility and/or 0% of reduction in motility.

### 4.9. Principal Components Analysis

The analysis of the main components (PCA) was carried out in order to establish if there was a correlation between the variables analyzed in this study. The variables were % of relative area for each compound, antimicrobial activity (inhibition zones) and percentage of motility inhibition; this analysis was carried out in order to find the minimum factors or components that provide us the maximum information about the variables involved.

### 4.10. Statistical Analysis

All experiments were performed in duplicate at least three times. Statistical analyses were performed with SPSS software (version 10.0, SPSS Inc., Chicago, IL, USA) and analyzed with an analysis of variance test; a post hoc test of Tukey’s HSD was used for the analyses of mean comparison. Differences between means were considered significant at *p*-values of ≤0.05. The multivariate analysis of principal component analysis (PCA) was performed using SPSS (Version 19, IBM Corp., and Chicago, IL, USA).

## 5. Conclusions

The antimicrobial activity of fractionated oregano is different for each fraction and will depend on the composition and amount of compounds present. Fraction 4, R, and P-OEO showed the best antimicrobial activity. The main compounds correlated with this antimicrobial activity are OM 2-5 (Terpinene-4-ol, Thymol, Carvacrol). Anti-motility activity was evidenced for all fractions with a varying percentage of inhibition, depending on the strain being tested. *P. aeruginosa* was the most resistant bacteria. Since F1 and F2 exhibited low antimicrobial activity yet demonstrated a strong anti-motility effect, this anti-motility activity appears to be influenced by the synergistic interaction of compound OM1 (Eucalyptol) with the other OM (2-5) compounds.

## Figures and Tables

**Table 1:** Physical properties of fractionated oregano.

Oil	Code	Color	Odor	Specific Gravity 20 °C g/mL
Fraction 1	F1	colorless	soft	0.842
Fraction 2	F2	intermediate yellow	intermediate	0.845
Fraction 3	F3	light yellow	intermediate	0.859
Fraction 4	F4	light brown	strong	0.884
Residual	R	strong brown	strong	0.512
Pure essential oil	P-OEO	light brown	intermediate	0.778

**Table 2:** Profile of the organic compounds found in the fractions analyzed by GC-MS.

COMPOSITION	Code	Relative Area ^1^ %					
		FI	F2	F3	F4	Residual	P-OEO
*o*-Cymene [20]	MH1	41.95	41.88	24.34	ND	ND	21.51
**α**-Phellandrene [20,25]	MH2	2.19	ND	ND	ND	ND	ND
**α**-Pinene [20,25]	MH3	1.45	ND	ND	ND	ND	ND
Camphene [25]	MH4	1.47	ND	ND	ND	ND	ND
**α**-Myrcene [6]	MH5	7.52	6.48	1.95	ND	ND	2.27
**α**-Phellandrene [6,25]	MH6	1.40	ND	ND	ND	ND	ND
Terpinolene [6]	MH7	5.52	5.32	2.35	ND	ND	ND
D-Limonene [20,25]	MH8	1.66	1.60	0.80	ND	ND	ND
Eucalyptol [20,25]	OM1	2.74	3.84	6.54	1.13	ND	4.57
γ-Terpinene [6,20,25]	MH9	26.77	27.32	19.35	ND	ND	12.75
Terpinen-4-ol [6,25]	OM2	ND	ND	1.28	1.55	ND	ND
Thymol [6,20]	OM3	1.59	1.92	9.86	21.17	10.42	13.53
Carvacrol [6,20,25]	OM4	3.48	4.36	22.37	60.23	48.54	34.09
**α**-Thujene [6,20,25]	MH10	2.19	1.30	ND	ND	ND	ND
Thymyl methyl ether	OM5	ND	ND	1.48	0.69	ND	ND
Caryophyllene [20,25]	SeH1	ND	ND	4.74	8.33	2.82	6.31
Humulene [20,25]	SeH2	ND	ND	1.78	4.70	3.84	2.90
*p*-Cymene-2,5-diol	OM6	ND	ND	ND	ND	1.61	ND
Phenol, 3-(1,1-dimethylethyl)-4-methoxy	OM7	ND	ND	ND	ND	9.58	ND
Caryophyllene oxide [25]	SeH3	ND	ND	ND	ND	11.07	ND
Humulene Epoxide II [6]	SeH4	ND	ND	ND	ND	4.39	ND
α-Terpinene [20]	MH11	ND	ND	ND	ND	ND	2.08
Monoterpene hydrocarbons	MH	92.12	83.91	48.79	0.00	0.00	38.60
Oxygenated monoterpenes	OM	7.81	10.12	41.53	84.77	70.14	52.19
Sesquiterpene hydrocarbons	SeH	0.00	0.00	6.52	13.03	22.13	9.21
Total components identified		99.92	94.03	96.85	97.79	92.27	97.79

^1^ Given as percentage of mean peak area from triplicate determination. ND = Not Detected. References: Pejčić et al. [6], Rostro- Alanís et al. [20], Asensio et al. [25].

**Table 3:** Antibiotic sensitivity of bacterial strains.

Antibiotic	Microorganisms		
	*P. aeruginosa*	*E. coli*	*P. vulgaris*
Amikacin (AK)	S *	S	S
Ampicillin (AM)	R	R	S
Cephalothin (CF)	R	R	S
Cefotaxime (CFX)	R	S	R
Ceftriaxone (CTX)	S	S	S
Chloramphenicol (CL)	S	S	R
Dicloxacillin (DC)	R	R	S
Gentamicin (GE)	S	S	S
Netilmicin (NET)	R	S	S
Nitrofurantoin (NF)	R	S	R
Penicillin (PE)	R	R	S
Sulfamethoxazole Trimethoprim (SXT)	R	S	R

* Susceptible (S), Resistant (R).

**Table 4:** Inhibition zones of fractionated *Poliomintha longiflora* essential oil.

Bacteria	Inhibition Zones (cm)						
	F1	F2	F3	F4	R	P-OEO	GE
*P. aeruginosa*	0.7 * ± 0.0 ^g^	NI	1.2 ± 0.2 ^ef^	1.4 ± 0.1 ^e^	1.0 ± 0.1 ^f^	0.7 ± 0.0 ^g^	1.7 ± 0.07 ^d^
*E. coli*	1.6 ± 0.3 ^de^	2.0 ± 0.3 ^cd^	2.9 ± 0.4 ^b^	3.8 ± 0.3 ^a^	2.6 ± 0.2 ^b^	3.3 ± 0.2 ^ab^	1.8 ± 0.1 ^d^
*P. vulgaris*	1.1 ± 0.1 ^f^	1.2 ± 0.2 ^ef^	2.3 ± 0.2 ^c^	2.4 ± 0.4 ^bc^	1.7 ± 0.3 ^d^	2.3 ± 0.4 ^c^	2.2 ± 0.2 ^c^

* Values of three repetitions ± standard deviation. Different letters between rows or columns (a–g) are significantly different. Fractions (F1–F4). Residual (R), Pure essential oil (P-OEO), positive control: Gentamicin (GE).

**Table 5:** Minimum bactericidal concentration (MBC).

Bacteria	MBC mg/mL						
	F1	F2	F3	F4	P-OEO	Residual	GE
*P. aeruginosa*	NI	NI	21.2 ± 2.5 ^a^	23.7 ± 2.5 ^a^	21.2 ± 2.5 ^a^	23.0 ± 2.5 ^a^	0.004 ± 0
*E. coli*	22.5 * ± 2.8 ^a^	2.1 ± 0.5 ^b^	0.1 ± 0.02 ^d^	0.002 ± 0.0 ^e^	0.002 ± 0.0 ^e^	<0.002	0.004 ± 0
*P. vulgaris*	1.1 ± 0.1 ^c^	1.0 ± 0.1 ^c^	0.1 ± 0.05 ^d^	0.002 ± 0.0 ^e^	0.2 ± 0.05 ^d^	0.2 ± 0.05 ^d^	0.002 ± 0

* Values of three repetitions ± standard deviation. Different letters between rows or columns (a–e) are significantly different. Fractions (F1–F4). Residual (R), Pure essential oil (P-OEO), positive control: Gentamicin (GE).

## Data Availability

The raw data supporting this article’s conclusions will be made avail-able, upon reasonable request, to the corresponding author.
